# Cannabis products and risk perceptions among people who consume and do not consume cannabis in the UK: findings from the International Cannabis Policy Study

**DOI:** 10.1186/s12954-026-01456-4

**Published:** 2026-04-24

**Authors:** Elle Wadsworth, Lindsey A. Hines, David Hammond, Tom P. Freeman

**Affiliations:** 1 Addiction and Mental Health Group, Department of Psychology, University of Bath, Bath, BA2 7AY UK; 2 RAND Europe, Cambridge, CB2 8BF UK; 3 School of Public Health Sciences, University of Waterloo, Waterloo, ON N2L 3G1 Canada

**Keywords:** Cannabis, Drug policy, Harm reduction, Risk perception, Method of administration

## Abstract

**Background:**

Population data on cannabis use—beyond prevalence and frequency of use—in the UK is lacking, yet needed to inform harm reduction messaging about cannabis. This study examined (a) recreational and medical cannabis use; (b) cannabis product use; (c) cannabis risk perceptions via three different routes of administration; (d) how product use and risk perceptions vary by reasons for use and higher risk use.

**Methods:**

Data were from UK respondents of the 2023 International Cannabis Policy Study. National surveys included 3444 UK residents and 1590 people who consumed cannabis in the past year aged 16–65. Weighted logistic regression examined associations between product use, risk perceptions and reasons for use.

**Results:**

50.9% of people who reported consuming cannabis reported doing so for recreational reasons only, 12.1% for medical reasons only, and 31.3% for both reasons. Dried flower was the most consumed product both in the past year (68%). Edibles (38.9%), vape oils (30.5%) and hash (27.5%) were the next commonly consumed products. People who consumed cannabis in the past year that screened positive for higher risk use were more likely to report consuming non-flower products (AOR 3.15, 95% CI 2.17, 4.58) than only dried flower. There were a higher percentage of respondents—regardless of whether they consumed cannabis or not—that reported perceiving consuming edibles daily as low or very low risk than smoking or vaping cannabis daily.

**Conclusions:**

There is diversity in the UK cannabis market in terms of the reasons for use and the products consumed. Understanding product use beyond just ‘cannabis’ and risk perceptions across different routes of administration is important for informing harm reduction messaging and evaluating policies to minimise harm and maximise benefit from both recreational and medical cannabis.

**Supplementary Information:**

The online version contains supplementary material available at 10.1186/s12954-026-01456-4.

## Introduction

Cannabis is the most widely consumed illegal substance in the UK. As of 2024, an estimated 6.8% of adults aged 16–59 and 13.8% of young adults (16–24 years) reported consuming cannabis in the past 12 months in England and Wales [1]. Cannabis potency and first-time treatment admissions for cannabis in addiction services have increased over the past two decades, as well as in Europe more broadly [2–5], indicating an important avenue to explore the relationships between what people are consuming (i.e., different products), how people are consuming (i.e., higher risk or problematic use) and the reasons why people consume cannabis (i.e., for medical purposes).

In the UK, cannabis commonly exists as dried flower (e.g., herbal or sinsemilla) or as resin (i.e., *hashish, hash*), is typically smoked and more often consumed alongside tobacco than not and has an average potency of 14.2% in most recent estimates [6, 7]. Previous research has shown that consumption in North American legal markets has diversified to non-smoked products [8–10]. An interesting dilemma is that while a shift to non-smoked products may be beneficial in terms of reducing smoke exposure and tobacco consumption, the non-smoked products tend to have greater potencies (e.g., vape oils, solid concentrates). Indeed, while dried flower tends to have a ceiling of around 30% tetrahydrocannabinol (THC), processed products using the extracts of the flower can reach much higher potencies [11]. Higher potency cannabis is a key public health concern given the association with increased risk of psychotic disorders and cannabis use disorders [12–17]. However, there are no data to examine the extent to which this product shift to higher potencies is happening in the UK, and no data on the relationship between product use and higher risk use in the UK beyond dried flower and hash [14, 18, 19].

Using an international convenience sample, authors used latent class analysis to examine different profiles of people who consume cannabis products and their association with higher risk use, using six types of cannabis: high potency dried flower without seeds, dried flower with seeds, hash, concentrates, kief, edibles [20]. The authors concluded that not only was there heterogeneity in people’s cannabis product use but that people consuming lower potency products had lower risk of cannabis dependence [20]. Indeed, the Lower-Risk Cannabis Use Guidelines (LRCUG) include recommendations to use lower potency products rather than higher potency products, as well as to avoid daily consumption and to avoid smoking cannabis, among other recommendations [21]. For harm reduction guidance to be effective in illegal markets such as the UK, it is critical to understand which products are available and their level of risk. This could enable people who consume cannabis in the UK to identify which products are higher risk, and empower them to make safer choices, if lower potency/lower risk products are currently available to them on the market, or to use less or not at all.

Previous research demonstrates that cannabis risk perceptions are associated with use, whereby decreases in risk perceptions are prospectively associated with increases in cannabis use and vice versa [22]. Where cannabis varies in terms of products, routes of administration and potencies, it is important to understand risk perceptions beyond overall cannabis and beyond ‘any’ use so that we can inform tailored harm reduction initiatives and drug policies to reduce harm. For instance, people who consume cannabis can smoke and vape multiple products of varying potencies at varying frequencies, e.g., smoke dried flower and hash or vape dried flower, oils, or concentrates, and harm reduction messaging may therefore differ accordingly. Studies examining the risk perceptions of different routes of cannabis administration have been conducted in North America, concluding that regular use of higher potency cannabis products and combustible cannabis products have greater risk perceptions (e.g., [23, 24]). However, to our knowledge, exploring risk perceptions of different routes of cannabis administration has not been conducted among a UK sample. With the cannabis product market diversifying, research is needed on the risk perceptions of different routes of cannabis administration to inform harm reduction messaging.

The legal context of cannabis—both medical or non-medical (recreational)—can determine the how people use cannabis, in terms of their own reasons for use (i.e., medical) or the product offerings available on the market. In the UK, unlicensed cannabis-based products for medical use became legal to prescribe in 2018, yet recreational cannabis remains illegal. National estimates of those consuming cannabis for medical reasons are unknown, yet only small numbers were reported to access legal prescribed cannabis in the first few years (n ~ 60) with illegal ‘medical’ use being more widespread [25, 26]. An issue with current estimates of cannabis use in the UK are that they come from crime surveys, e.g., the Crime Survey for England and Wales, which does not distinguish between recreational use and medical use, the latter of which may be legal and not a crime [27]. The scale with which people use cannabis for medical and/or recreational cannabis use in the UK, and whether products used, routes of administration, and risk perceptions vary between medical or recreational purposes is unclear yet may be critical in tailoring harm reduction messaging. Little research has been conducted in the UK that examines cannabis use among people who consume cannabis for recreational and medical reasons, yet what little research exists points to individual differences in recreational and medical consumer profiles that may be important for clinical practice [28].

To our knowledge, this is the first study to examine cannabis product use and risk perceptions among people who consume cannabis both for medical and recreational reasons in the UK. The aims of the study were fourfold: (1) to examine the breakdown of people who consume cannabis for recreational and medical reasons; (2) to examine the different cannabis products used among respondents who had consumed cannabis in the past 12 months; (3) to examine the risk perceptions of consuming cannabis daily via different routes of administration (e.g., smoking, vaping, eating) among all respondents; and (4) to examine how cannabis product use and risk perceptions vary across reasons for use and higher risk use among people who consume cannabis.

## Methods

Data were from the 2023 of the International Cannabis Policy Study (ICPS), which consists of national, repeat cross-sectional surveys conducted annually in Canada, United States, Australia, New Zealand, UK and Germany. The current study reported data from the UK sample only. Data were collected via self-completed web-based surveys in September–October in 2023 from respondents aged 16–65. A non-probability sample of respondents was recruited through the Nielsen Consumer Insights Global Panel and their partners’ panels. Nielsen draws stratified random samples from the online panels, with quotas based on sex and age. Respondents aged 16 and 17 required parental consent before accessing the survey. For the UK sample, people who consumed cannabis in the past 12 months were oversampled to ensure sufficient power for analyses among people who consume cannabis. Upon completion, respondents received remuneration in accordance with their panel’s usual incentive structure. The American Association for Public Opinion Research (AAPOR) cooperation rate [29], which is the percentage of respondents who completed the survey among all eligible respondents who accessed the survey link, was 55.1% in 2023. UK surveys were conducted in English and the median survey time was 22 min.

The study was reviewed by and received ethics clearance through the University of Waterloo (ORE#31330) and the University of Bath (0513-586). A full description of the study methods can be found in the ICPS Technical Reports and methodology paper [30, 31]. The analysis plan for this paper was pre-registered on the Open Science Framework prior to data analysis (https://osf.io/jh7az/).

### Measures

*Socio-demographic measures.* Sex-at-birth, gender, age, ethnicity/race, highest education level, perceived income adequacy, and region (Table 1). For “perceived income adequacy” and “race/ethnicity” those who answered, “Don’t know” or “Refuse to answer” were categorized to “Unstated”. In regression models, respondents identifying as ‘Other’ or ‘Unstated’ were classed as missing due to insufficient cell counts. For region, regions within England but not London were combined in regression models. Both gender and sex-at-birth were collected separately in the ICPS; however, sex-at-birth was included in models to retain all respondents as gender included missing data and very small cell sizes for non-cis individuals, which could not be included in analyses but are shown in Table 1.

**Table 1:** Sample characteristics among all UK respondents (n = 3444) and UK respondents who reported using cannabis in the past 12 months, 2023 (n = 1590)

Age	All respondents (n = 3444)		Respondents who reported using cannabis in the past 12 months (n = 1590)	
	Unweighted % (n)	Weighted % (n)	Unweighted % (n)	Weighted % (n)
16–25	23.1 (796)	17.6 (606)	27.2 (432)	37.5 (596)
26–35	17.2 (592)	21.9 (755)	18.1 (287)	19.7 (313)
36–45	26.9 (925)	20.2 (694)	31.4 (500)	22.6 (359)
46–55	16.5 (567)	20.9 (718)	11.7 (186)	10.5 (168)
56–65	16.4 (564)	19.5 (670)	11.6 (185)	9.8 (155)
*Sex at birth*				
Female	51.0 (1756)	50.8 (1748)	51.9 (826)	39.7 (631)
Male	49.0 (1688)	49.2 (1696)	48.1 (764)	60.3 (959)
*Gender*				
Woman	50.7 (1747)	50.3 (1733)	51.3 (816)	39.5 (628)
Man	48.2 (1660)	48.7 (1677)	47.5 (756)	59.4 (945)
Other	0.4 (11)	0.3 (9)	0.3 (5)	0.3 (5)
Unstated	0.8 (26)	0.7 (24)	0.8 (13)	0.7 (12)
*Race/ethnicity*				
Asian or Asian British	6.3 (218)	9.1 (313)	4.7 (75)	6.3 (100)
Black, Black British, Caribbean or African	3.7 (129)	4.6 (159)	3.3 (53)	5.7 (91)
Mixed or multiple ethnic groups	3.9 (133)	2.9 (101)	5.2 (83)	6.0 (96)
White	84.5 (2909)	81.8 (2816)	85.3 (1357)	80.5 (1280)
Other/Unstated	1.6 (55)	1.6 (55)	1.4 (22)	1.5 (24)
*Highest level of education*				
Less than high school	13.9 (465)	22.5 (756)	12.5 (194)	20.9 (325)
High school diploma	16.2 (542)	14.3 (482)	15.6 (241)	18.8 (292)
Some college or technical vocation	34.5 (1159)	34.9 (1173)	34.6 (535)	33.3 (516)
Bachelor’s degree or higher	35.5 (1190)	28.3 (954)	37.3 (578)	26.9 (418)
*Income adequacy*				
Very difficult or difficult	26.7 (921)	30.2 (1041)	24.7 (392)	26.5 (422)
Neither easy nor difficult	33.7 (1162)	36.0 (1240)	31.3 (497)	33.1 (526)
Very Easy or easy	36.9 (1270)	31.0 (1069)	41.8 (665)	38.1 (606)
Unstated	2.6 (91)	2.7 (95)	2.3 (36)	2.3 (36)
*Region*				
North East	4.3 (149)	3.9 (133)	4.0 (64)	4.3 (69)
North West	11.6 (398)	11.0 (377)	11.1 (176)	10.3 (164)
Yorkshire and the Humber	7.3 (250)	8.1 (279)	7.0 (112)	8.3 (133)
East Midlands	6.3 (218)	7.1 (243)	5.6 (89)	5.3 (84)
West Midlands	9.0 (308)	8.9 (304)	8.7 (138)	8.2 (130)
East of England	8.8 (302)	9.3 (321)	7.4 (118)	6.7 (107)
London	19.8 (680)	14.3 (490)	28.4 (451)	25.9 (412)
South East	11.7 (401)	13.7 (470)	9.1 (144)	9.8 (156)
South West	6.8 (234)	8.2 (282)	5.3 (84)	5.1 (82)
Wales	4.0 (139)	4.5 (155)	3.4 (54)	3.5 (56)
Scotland	7.9 (272)	8.3 (286)	7.7 (123)	9.7 (156)
Northern Ireland	2.6 (90)	2.8 (97)	2.3 (37)	2.8 (44)
*Cannabis use frequency*				
Never	35.3 (1217)	58.1 (2002)	–	
Consumed cannabis more than 12 months ago	18.5 (637)	34.7 (1197)	–	
Less than monthly, but in the past 12 months	14.4 (496)	2.1 (73)	31.2 (496)	29.7 (473)
Monthly	11.4 (391)	1.8 (63)	24.6 (391)	25.5 (405)
Weekly	8.0 (275)	1.2 (43)	17.3 (275)	17.3 (275)
Daily or near daily	12.4 (428)	2.0 (68)	26.9 (428)	27.5 (437)
*Reasons for cannabis use*				
Medical use only	–	–	13.4 (209)	12.1 (188)
Recreational use only	–	–	39.3 (771)	50.9 (793)
Both medical and recreational use	–	–	31.0 (485)	31.3 (488)
Don’t know	–	–	6.3 (99)	5.8 (90)
*Higher risk use (CUDIT-SF)*				
Screened positive (score of 2 or higher)	–	–	43.4 (690)	45.0 (715)
Screened negative	–	–	56.6 (900)	55.0 (875)

*Cannabis use frequency.* Cannabis use frequency was assessed using three questions: “Have you ever tried cannabis?”, “How often do you use cannabis?” and “When was the last time you used cannabis?” Responses were categorized as “Never”, “Used more than 12 months ago”, “Less than monthly but used in the past 12 months”, “Monthly”, “Weekly”, and “Daily/near daily”. For analyses, categories were grouped to “Never consumed cannabis or consumed more than 12 months ago” and “Consumed in the past 12 months” (Less than monthly/Monthly/Weekly/Daily).

*Cannabis product use in past 12 months.* Respondents who consumed cannabis in the past 12 months were asked: “Have you used marijuana in any of the following ways?” for (1) Dried flower (smoked or vaped, including pre-rolled joints); (2) Cannabis oils or liquids taken orally (e.g., drops, capsules or sprays); (3) Cannabis oils or liquids for vaping; 4) Edibles/foods; (5) Drinks (e.g., cannabis cola, tea or coffee); (6) Concentrates (e.g., wax, shatter, budder, resin, rosin, crumble); (7) Hash or kief; (8) Tinctures (concentrated amounts ingested orally or taken under the tongue); (9) Topical ointments (e.g., skin lotions or bath products). Response options were “No”, “Yes but NOT in the past 12 months”, “Yes in the past 12 months”, “Don’t know”. Responses were categorised into a binary variable: “Yes” (Yes in the past 12 months), “No” (No, Yes but NOT in the past 12 months, Don’t know). For regression analyses, responses were categorised into a binary variable of “dried flower use only” versus “non-flower product use” (which includes multiple product use).

*Cannabis product use frequency in past 12 months.* Respondents who reported using a specific product in the past 12 months were asked “In the past 12 months, how often did you use [product].” Responses were “Less than once a month”, “Monthly”, “Weekly”, “Daily”, “Don’t know”. Responses were categorised into a binary variable: “Daily”, “Other”.

*Average amount of dried flower consumed per day on days used.* Respondents who consumed dried flower in the past 12 months were asked about the quantity consumed on the last day they used dried flower. Full measures used and cleaning process is described elsewhere [9].

*Reasons for cannabis use.* Respondents who consumed cannabis in the past 12 months were asked: “Do you use cannabis for medical reasons, recreational reasons or both? By medical cannabis user, we mean someone who uses cannabis only to manage a medical condition.” Response options were “Medical use only”, “Recreational use only”, “Both recreational and medical use”, “Don’t know”. For models including people who had not consumed cannabis in the past 12 months, an additional level was created “Non-consumer”. For all models, “Don’t know” was removed.

*Higher risk use.* Higher risk use was assessed using the Cannabis Use Disorder Identification Test Revised Short Form (CUDIT-SF) [32]. The CUDIT-SF is a 3-item form that determines a positive screen to be a score 2 and above. This positive screen threshold showed good discrimination of DSM-5 Cannabis Use Disorder, with an area under the curve exceeding 0.8 in two independent samples. Respondents who consumed cannabis in the past 12 months were asked the following three questions: (1) “How often during the past 6 months did you find that you were not able to stop using cannabis once you had started?”; (2) “How often during the past 6 months have you devoted a great deal of your time to getting, using, or recovering from cannabis?”; and (3) How often in the past 6 months have you had a problem with your memory or concentration after using cannabis?” All with response options “Never”, “Less than monthly”, “Monthly”, “Weekly”, “Daily or almost daily”. One measure was created from the three questions, where “Never” = 0, “Less than monthly” = 1, “Monthly” = 2, “Weekly” = 3, and “Daily or almost daily” = 4. Respondents with a total of 2 or more were categorised into “Positive screen” and those with a total of 0 or 1 were categorised into “Negative screen”.

*Perceived risk of daily cannabis product use.* All respondents were asked: “In your opinion, what is the level of health risk from…” (1) smoking cannabis daily; (2) vaping cannabis daily; (3) consuming cannabis edibles daily. Respondents could respond with options “Very low risk”, “Low risk”, “Moderate risk”, “High risk”, “Very high risk” and “Don’t know”. To focus on respondents with low levels of perceived risk, responses were categorised into a binary variable in models: “Very/low risk” and “Other” (Moderate, High, Very high, Don’t know).

All questions included “Don’t know” and “Refuse to answer” options and were excluded unless specified within the measures above.

### Analysis

A total of 4010 UK respondents completed the 2023 survey. After removing respondents due to self-reported dishonesty (n = 78) determined by asking participants “Were you able to provide ‘honest’ answers about your cannabis use during the survey?” and those who selected “No” or “For some questions, but not all” were excluded; poor data quality determined by those who did not select the current month when asked (n = 227); those who identified as intersex and an ‘other’/unstated sex (due to insufficient cell counts for weighting) (n = 3);’ speeding (n = 20); duplicate entries (n = 223); and unstated region/state/territory (n = 15), 3444 respondents were retained. The 3444 comprised the 2023 analytic sample. A sub-sample of respondents who consumed cannabis in the past 12 months (n = 1590) were explored in the current analysis. Missing data were removed using case-wise deletion for variables used in regression models: highest level of education (n = 88); race/ethnicity (n = 55); region (n = 3); reasons for cannabis use (n = 125); perceived risk of smoking cannabis daily (n = 21); vaping cannabis daily (n = 21); and consuming cannabis edibles daily (n = 20).

Post-stratification sample weights were constructed to calibrate to known population proportions as available in censuses and national benchmark surveys. Respondents were classified into age-by-sex-by-region groups, ethnicity-by-region groups, education groups, and age-by-sex-by-cannabis-use groups. Correspondingly grouped population proportion estimates were obtained from national government agencies [33–41]. A raking algorithm was applied to compute weights that are calibrated to these groupings. The SAS macro “RAKE_AND_TRIM_G4_V5” was used, with trimming to 5 (rescaled) if necessary. Finally, the weights were rescaled to sum to the sample size in the country. Estimates are weighted unless otherwise specified.

First, descriptive statistics were used to describe the percentage of respondents consumed each of the 10 different cannabis products, both for use in the past 12 months and daily. Second, descriptive statistics were used to describe the risk perceptions of daily use of three different cannabis routes of administration (smoking, vaping, consuming edibles) by their reason for cannabis use (medical, recreational, both). Third, binary logistic regression models were used to fit univariable and multivariable estimates of the associations between the use of cannabis products (dried flower only versus non-flower products with or without dried flower) and (1) reasons for use and (2) higher risk cannabis use among people who consume cannabis only. Dried flower was separated in the variable as the most commonly used product. Finally, binary logistic regression models were used to fit univariable and multivariable estimates of the association between risk perceptions of different routes of administration and reasons for use (medical, recreational, both, non-consumer) among all respondents.

Post-hoc analyses that were not pre-registered were conducted on (1) the associations between risk perceptions of different routes of cannabis administration and frequency of cannabis use among people who consume cannabis using univariable and multivariable binary logistic regression models; and (2) the average amount of dried flower in grams consumed in a session among people who consume cannabis.

All models were adjusted for region, age groups, sex-at-birth, highest level of education, ethnicity/race, and income adequacy. Adjusted odds ratios (AOR) are reported with 95% confidence intervals (95% CI). Analyses were conducted using R version 4.4.1, with survey weighting and svyglm for models.

## Results

Table 1 displays the sample characteristics of UK respondents and UK respondents who reported using cannabis in the past 12 months in 2023. Across all respondents, 50.8% were female-at-birth, 39.5% were aged 35 and under, and 81.8% identified as White. Among respondents who reported using cannabis in the past 12 months, 39.7% were female-at-birth, 57.2% were aged 35 and under, 80.5% identified as White, and 27.5% reported using cannabis daily or near daily. Fifty one percent of people who consumed cannabis reported using cannabis for recreational reasons only, 12.1% for medical reasons, 31.3% for both recreational and medical reasons, and 5.8% reported that they did not know. 45.0% of people who consumed cannabis screened positive for higher risk use using the CUDIT-SF.

### Cannabis product use in the past 12 months

**Fig. 1:**
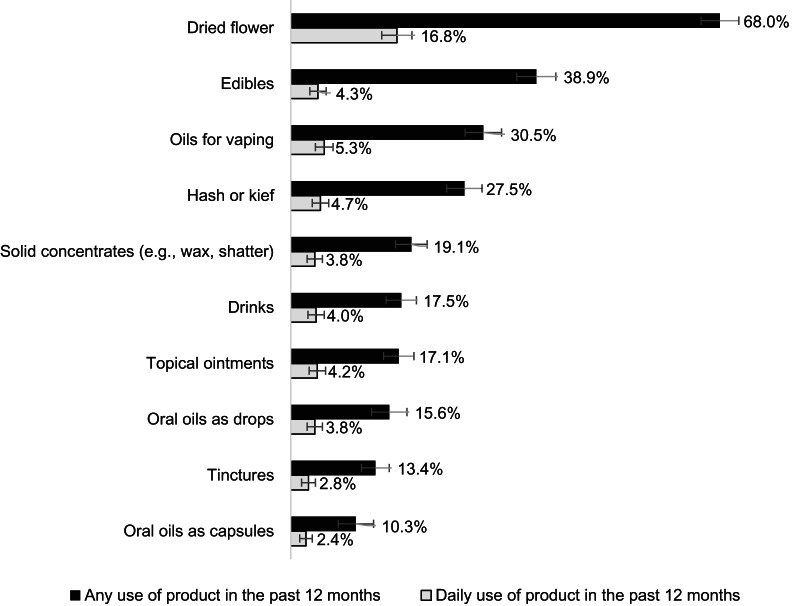
Weighted percentage of different product consumption in the past 12 months and daily (n = 1590)

Figure 1 presents the percentage of respondents who consumed cannabis in the past 12 months who reported using different products in the past 12 months and the percentage who reported using that product daily. Dried flower was the most consumed product both in the past 12 months (68.0%). Edibles (38.9%), vape oils (30.5%) and hash (27.5%) were the next commonly consumed product in the past 12 months, with tinctures (13.4%) and oral capsules (10.3%) as the least consumed product.

Table 2 displays binary logistic regression analyses of past 12-month use of cannabis products: non-flower products (with or without dried flower) versus dried flower only. Reasons for cannabis use was associated with dried flower/non-flower product use. Respondents who consumed cannabis for medical reasons only had higher odds of reporting the use of non-flower products in the past 12 months than respondents using cannabis for recreational reasons only and respondents using cannabis for both recreational and medical reasons. Higher risk use as measured by the CUDIT-SF was associated with non-flower product use. Respondents who screened positively on the CUDIT-SF had higher odds of reporting non-flower product use in the past 12 months than respondents who did not screen positively. Individuals aged 16–45, educated to a Bachelors degree or higher, and reported to find making ends meet very easy/easy had higher odds of reporting the use of non-flower products in the past 12 months.

**Table 2:** Binary logistic regression models of the associations between different cannabis product use, reasons for cannabis use, and higher risk use (n = 1428)

	Non-flower products (with or without dried flower) versus dried flower only	
	Univariable analysis	Multivariable analysis
	OR (95% CI)	AOR (95% CI)
*Reasons for use*		
Recreational only (vs. medical only)	**0.39 (0.23, 0.64)**	**0.30 (0.18, 0.52)**
Medical only (vs. both)	**1.77 (1.03, 3.05)**	**2.30 (1.29, 4.11)**
Both (vs. recreational only)	**1.47 (1.05, 2.06)**	1.43 (0.99, 2.05)
*CUDIT-SF* *(vs. Negative screen)*		
Positive screen	**3.17 (2.21, 4.54)**	**3.15 (2.17, 4.58)**
*Region* *(vs. England excl. London)*		
London		1.23 (0.79, 1.94)
Wales		0.46 (0.15, 1.44)
Scotland		1.30 (0.69, 2.47)
Northern Ireland		2.45 (0.86, 7.01)
*Sex-at-birth* *(vs. Female)*		
Male		1.18 (0.85, 1.64)
*Age* *(vs. 56–65)*		
16–25		**1.77 (1.07, 2.94)**
26–35		**2.10 (1.14, 3.88)**
36–45		**1.86 (1.08, 3.19)**
46–55		1.21 (0.69, 2.13)
*Ethnicity/race* *(vs. White)*		
Asian		1.42 (0.67, 2.99)
Black		1.03 (0.42, 2.50)
Mixed		0.96 (0.44, 2.10)
*Highest level of education* *(vs. Less than high school)*		
Completed high school		1.06 (0.62, 1.82)
Some college or technical vocation		1.27 (0.78, 2.07)
Bachelor’s degrees or higher		**2.09 (1.26, 3.45)**
*Income adequacy* *(vs. very difficult/difficult)*		
Neither easy nor difficult		1.42 (0.94, 2.14)
Very Easy/Easy		**1.94 (1.27, 2.96)**
Unstated		2.18 (0.66, 7.11)

Bold values are statistically significant at the 5% level

Supplemental Table 1 displays the average grams of dried flower consumed per day, on days used (reported as either joints or g/oz), among people who consume dried flower. The mean grams of dried flower consumed per day was 1.65 g (95% CI 1.53, 1.78). The median grams of dried flower consumed per day was 1.00 g, with 0.40 g at the 25th quantile and 2.40 g at the 75th quantile. The average amount of dried flower consumed per day on days used was greater among people who consumed daily than those who did not consume daily.

### Risk perceptions of different routes of daily cannabis administration

**Fig. 2:**
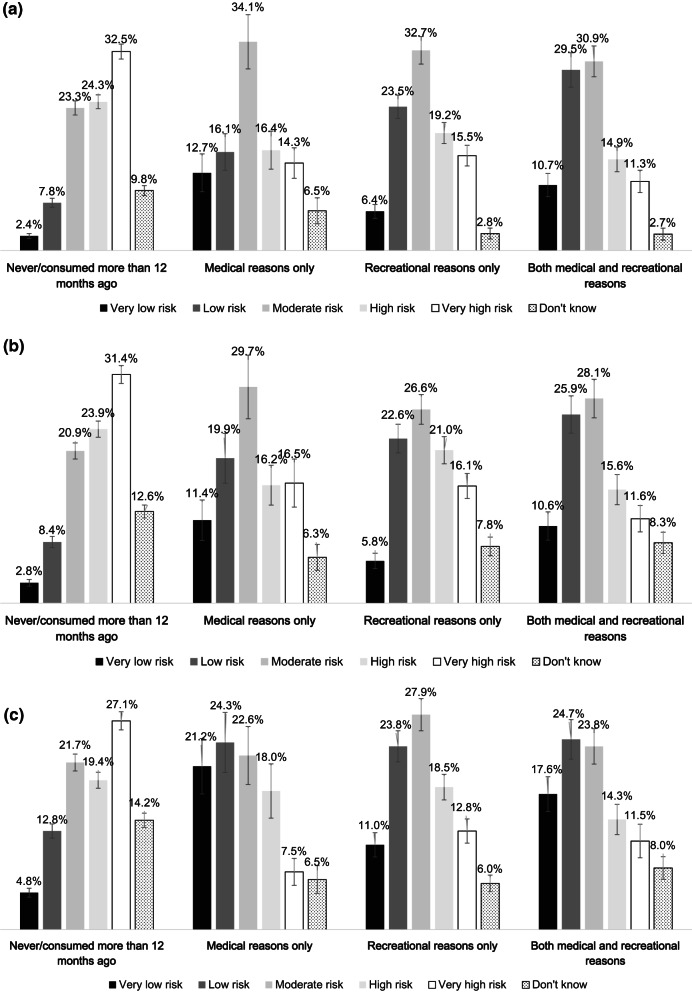
**a** Risk perceptions of *smoking cannabis daily* by reasons for cannabis use, 2023 (n = 3444). **b** Risk perceptions of *vaping cannabis daily* by reasons for cannabis use, 2023 (n = 3444). **c** Risk perceptions of *consuming cannabis edibles daily* by reasons for cannabis use, 2023 (n = 3444)

Figure 2a–c display the risk perceptions of different routes of cannabis administration among people who do and do not use cannabis in 2023. For risk perceptions of smoking cannabis daily (Fig. 2a), 10.2% of those who reported not consuming cannabis in the past 12 months, 28.8% of those consuming cannabis for medical reasons, 29.9% of those consuming cannabis for recreational reasons only, and 40.2% of those consuming cannabis for both recreational and medical reasons reported perceiving smoking cannabis daily to be low or very low risk.

For risk perceptions of vaping cannabis daily (Fig. 2b), 11.2% of those who reported not consuming cannabis in the past 12 months, 31.3% of those consuming cannabis for medical reasons, 28.4% of those consuming cannabis for recreational reasons only, and 36.5% of those consuming cannabis for both recreational and medical reasons reported perceiving vaping cannabis daily to be low or very low risk.

For risk perceptions of consuming edibles daily (Fig. 2c), 17.6% of those who reported not consuming cannabis in the past 12 months, 45.5% of those consuming cannabis for medical reasons, 34.8% of those consuming cannabis for recreational reasons only, and 42.3% of those consuming cannabis for both recreational and medical reasons reported perceiving consuming cannabis edibles daily to be low or very low risk.

Table 3 displays binary logistic regression analyses of risk perceptions of three different routes of daily cannabis administration. Reasons for cannabis use was associated with the risk perceptions of all three routes of administration. Respondents who consumed cannabis for any reasons had higher odds of perceiving smoking, vaping and consuming cannabis edibles daily as low or very low risk than respondents who do not consume cannabis. Respondents who consumed cannabis for recreational reasons only had lower odds of reporting a perception that smoking cannabis daily and vaping cannabis daily was low or very low risk compared to respondents who consumed cannabis for both medical and recreational reasons and lower odds of reporting a perception that consuming edibles daily was low or very low risk compared to respondents who consumed cannabis for medical reasons only.

**Table 3:** Binary logistic regression models of the associations between risk perceptions of different routes of cannabis administration and reasons for cannabis use

	Smoking cannabis daily Very low/low risk (vs. Else) (n = 3199)		Vaping cannabis daily Very low/low risk (vs. Else) (n = 3199)		Consuming cannabis edibles daily Very low/low risk (vs. Else) (n = 3201)	
	Univariable analysis	Multivariable analysis	Univariable analysis	Multivariable analysis	Univariable analysis	Multivariable analysis
	OR (95% CI)	AOR (95% CI)	OR (95% CI)	AOR (95% CI)	OR (95% CI)	AOR (95% CI)
*Reasons for use*						
Medical only (vs. non-consumer)	**3.58 (2.36, 5.43)**	**3.70 (2.38, 5.75)**	**3.60 (2.40, 5.40)**	**3.71 (2.37, 5.80)**	**3.90 (2.68, 5.68)**	**4.27 (2.86, 6.37)**
Medical only (vs. recreational only)	0.94 (0.62, 1.45)	1.11 (0.70, 1.75)	1.15 (0.75, 1.74)	1.35 (0.85, 2.15)	**1.56 (1.05, 2.33)**	**1.71 (1.11, 2.61)**
Medical only (vs. both)	**0.60 (0.38, 0.94)**	0.69 (0.43, 1.11)	0.79 (0.51, 1.23)	0.92 (0.57, 1.48)	1.14 (0.75, 1.73)	1.28 (0.83, 1.99)
Recreational only (vs non-consumer)	**3.78 (2.90, 4.92)**	**3.34 (2.45, 4.55)**	**3.14 (2.41, 4.11)**	**2.74 (2.01, 3.74)**	**2.49 (1.96, 3.17)**	**2.50 (1.89, 3.30)**
Recreational only (vs. both)	**0.63 (0.47, 0.86)**	**0.62 (0.44, 0.87)**	**0.69 (0.51, 0.95)**	**0.68 (0.49, 0.94)**	**0.73 (0.58, 0.93)**	0.75 (0.55, 1.03)
Both (vs. non-consumer)	**5.96 (4.41, 8.04)**	**5.36 (3.87, 7.43)**	**4.54 (3.37, 6.11)**	**4.04 (2.92, 5.58)**	**3.43 (2.62, 4.51)**	**3.33 (2.47, 4.47)**
*Region* *(vs. England, excl. London)*						
London		1.17 (0.73, 1.87)		1.09 (0.69, 1.72)		0.71 (0.46, 1.08)
Wales		0.71 (0.32, 1.57)		0.96 (0.46, 2.00)		0.84 (0.44, 1.60)
Scotland		1.42 (0.82, 2.47)		0.87 (0.50, 1.52)		1.20 (0.75, 1.91)
Northern Ireland		1.39 (0.59, 3.27)		0.45 (0.15, 1.32)		1.18 (0.53, 2.63)
*Sex-at-birth* *(vs. Female)*						
Male		1.14 (0.83, 1.57)		1.32 (0.98, 1.79)		1.26 (0.98, 1.63)
Age (vs. 56–65)						
16–25		**2.29 (1.29, 4.08)**		**2.14 (1.22, 3.77)**		1.04 (0.66, 1.63)
26–35		**2.97 (1.72, 5.13)**		**2.50 (1.47, 4.26)**		1.15 (0.75, 1.75)
36–45		**2.23 (1.31, 3.79)**		**2.14 (1.30, 3.53)**		1.29 (0.89, 1.87)
46–55		1.06 (0.58, 1.92)		1.31 (0.77, 2.23)		0.94 (0.63, 1.39)
*Ethnicity/race* *(vs. White)*						
Asian		0.94 (0.49, 1.83)		0.76 (0.40, 1.44)		0.73 (0.41, 1.29)
Black		0.65 (0.30, 1.40)		1.00 (0.47, 2.15)		1.19 (0.60, 2.36)
Mixed		**0.37 (0.21, 0.66)**		0.47 (0.15, 1.45)		0.55 (0.23, 1.31)
*Highest level of education* *(vs. Less than high school)*						
Completed high school		1.39 (0.79, 2.42)		1.03 (0.61, 1.75)		1.24 (0.79, 1.94)
Some college or technical vocation		1.11 (0.68, 1.80)		0.99 (0.63, 1.57)		1.03 (0.70, 1.52)
Bachelor’s degrees or higher		0.89 (0.53, 1.49)		0.80 (0.48, 1.32)		0.93 (0.62, 1.41)
*Income adequacy* *(vs. very difficult/difficult)*						
Neither easy nor difficult		0.97 (0.64, 1.45)		0.97 (0.65, 1.43)		**0.65 (0.47, 0.90)**
Very easy/Easy		0.80 (0.53, 1.20)		0.93 (0.63, 1.39)		0.80 (0.58, 1.10)
Unstated		0.43 (0.12, 1.52)		0.62 (0.18, 2.12)		**0.34 (0.12, 0.99)**

Bold values are statistically significant at the 5% level

Supplemental Table 2 displays binary logistic regression analyses of risk perceptions of three different routes of daily cannabis administration by frequency of use among people who consumed cannabis. Respondents who consumed cannabis daily had higher odds of perceiving smoking, vaping and consuming cannabis edibles daily as low or very low risk than respondents who consumed cannabis less than monthly. Respondents who consumed cannabis weekly had higher odds of perceiving smoking and vaping daily as low or very low risk than respondents who consumed cannabis less than monthly. Respondents who consumed cannabis monthly had higher odds of perceiving vaping and consuming edibles daily as low or very low risk than respondents who consumed cannabis less than monthly.

## Discussion

While cannabis can be legally prescribed for medical use in the UK, recreational cannabis remains illegal. A key finding from this study is that half of people who consumed cannabis in the past year in the UK reported doing so for recreational reasons only. Looking at medical reasons only, this was reported by 12% of people who reported past-year cannabis use or 1% of all UK respondents. When we included those who reported using cannabis for both medical and recreational reasons, this percentage increased to 43.4% of people who reported past-year cannabis use or 3% of UK respondents. There are little existing data detailing the prevalence or percentage of people who consume cannabis for medical purposes in the UK. However, a study exploring the extent of the illegal medical cannabis market in the UK estimated that a similar percentage to the current study—close to 3% of UK residents were using cannabis to relieve their symptoms [25]. Similar to that study’s conclusions [25], with the medical cannabis prescriptions not totalling the number of respondents reportedly using cannabis for medical reasons [26], our findings suggest that there is a substantial and potentially underserved community who are self-medicating via the illegal market and without support of a healthcare professional.

Dried flower was the most consumed product both in the past 12 months and daily, but while dried flower remains the most consumed product, it was not the only product used in the UK. Our results confirm that people who consume cannabis in the UK are consuming a diverse offering of products, including dried flower, vape oils, edibles, solid concentrates, oral oils, hash, and topicals, and this diversity of products consumed is also seen in international samples in both legal and illegal markets [9, 20]. As the UK cannabis market offers more than just dried flower and hash, future research in the UK should expand beyond just examining ‘cannabis’. It is worth highlighting that hash is traditionally one of the dominant products in Europe alongside dried flower [42], yet hash ranked fourth in the current study for past year use, behind edibles and vape oils. Considering that these non-flower products are typically higher in potency, harm reduction advice for people who consume cannabis should be tailored to different products, such as recommending using lower potency over higher potency products [21]. Indeed, non-flower processed products can be manufactured to various potencies, including lower potencies than dried flower, so while non-flower products tend to be of higher potency, they also offer potential for dose control that may not be available with dried flower from illegal markets.

People screening positive for higher risk cannabis use were more likely to report consuming non-flower products in the past 12 months, products which typically contain higher potencies than dried flower. This finding aligns with previous research among UK and international populations, whereby higher potency cannabis had an association with increased severity of dependence or higher risk use [14, 18–20] and extends previous research to specifically account for these products consumed by people in the UK. In contrast, people screening positive for higher risk use were less likely to report consuming only dried flower in the past 12 months. This is perhaps more of an indication of the popularity of dried flower among people who consume cannabis at a lower intensity and not partake in multiple products or other higher potency products. A similar finding was observed in a nationally representative sample of people who consume cannabis in the U.S., whereby those screening for cannabis use disorder were less likely to report smoking cannabis [43]; however, it is important to note that the current study examined dried flower use, which can also be consumed in ways other than smoking. Similar to what was highlighted earlier, this association between higher potency products and increased risk of higher risk use could point to a clinical need for discussing the specific products consumed and to provide harm reduction advice for non-flower products that are typically higher in potency. This could be further tailored to people who consume cannabis for medical reasons, as findings from this study demonstrated they were more likely to consume non-flower products. The relationship between medical cannabis use, non-flower products and higher risk use warrants further exploration.

In general, there were a higher percentage of UK respondents—regardless of whether they consumed cannabis or not—that reported perceiving consuming edibles daily as low or very low risk than smoking or vaping cannabis daily. However, the percentages varied considerably between categories, whereby close to half of people who consumed cannabis for medical reasons only perceived consuming edibles daily to be low/very low risk compared to only one in ten people who did not consume cannabis in the past 12 months. Perceiving edibles as lower risk than smoking is supported by previous literature, where perhaps the non-combustible method of administration feeds into the reduced perception of harm [23, 44, 45] and aligns with the recommendation in the LRCUG of avoiding smoking cannabis as a route of administration [21]. Yet, edibles are not without harm. While they do not have the harm associated with inhalation, they present a risk of accidental over-consumption due to their delayed onset of psychoactive effects typical of oral consumption combined with their potential to contain higher potencies [44, 46].

Respondents who consumed cannabis in the past 12 months were more likely to report perceiving smoking, vaping or consuming edibles daily to be low or very low risk than those who do not consume cannabis, and those consuming cannabis more frequently (e.g., daily) were more likely to report perceiving smoking vaping or consuming edibles daily to be low risk than those consuming cannabis less frequency (e.g., less than monthly), aligning with previous literature on perceptions of harm from cannabis generally (e.g., [47]). Respondents who reported consuming cannabis for both recreational and medical reasons were more likely to report low/very low risk perceptions of smoking or vaping cannabis daily and respondents who consumed cannabis for medical reasons were more likely to report low/very low risk perceptions of consuming edibles daily. Previous research supports the current finding that people who consume cannabis for medical reasons have lower risk perceptions than those who consume recreationally due to their intention of consumption [43, 48, 49]. Moreover, edibles that can be consumed daily may be seen as a more ‘medical’ product as they are available in the legal UK medical market, i.e., gummies or pastilles (e.g., [50]). Whereas the edibles that may be consumed more recreationally (e.g., cookies, cakes), may not be seen as a product for daily consumption, whether that be homemade or purchased [51]. Reiterating what was mentioned above, harm reduction messaging should be tailored to people who consume medical cannabis and consume frequently to emphasise the potential harms and benefits from products used by this population, particularly non-flower products such as edibles.

### Limitations

This study is subject to limitations common to survey research. Respondents were recruited using non-probability-based sampling; therefore, the findings do not necessarily provide nationally representative estimates. However, the data were weighted by age group, sex, region-by-ethnicity, education, and cannabis use status to represent the UK population. Self-reported data are subject to social desirability biases. At the time of the study, recreational cannabis was illegal in the UK; therefore, patterns of cannabis use may be underreported or misrepresented. However, ICPS surveys include several measures to promote honest reporting, including assurances of confidentiality and impartiality statements.

## Conclusions

The findings from this study demonstrate that there is diversity in the UK cannabis market in terms of the reasons for use (i.e., recreational, medical) and the products consumed. The products consumed in the UK extend beyond dried flower, with between one quarter and one third of people who reported using cannabis using edibles, vape oils or hash in the past 12 months. We recommend that points of contact with people who consume cannabis—i.e., medical cannabis prescribers or treatment services—should include a discussion of product use beyond just cannabis, tailoring harm reduction messaging to minimise harm and maximise benefit from both recreational and medical cannabis use in the UK.

## Supplementary Information

Supplementary Material.

## Data Availability

The data that support the findings of this study are available from the International Cannabis Policy Study (www.cannabisproject.ca) but restrictions apply to the availability of these data and so are not publicly available. Data are however available from the authors upon reasonable request.
