# Supplementary material for: Cannabis products and risk perceptions among people who consume and do not consume cannabis in the UK: findings from the International Cannabis Policy Study
Source: Harm Reduct J. 2026 Apr 24;23:104. doi: 10.1186/s12954-026-01456-4 (PMC13262506; doi:10.1186/s12954-026-01456-4)
Supplement: Supplementary file 1 — Supplementary Material. [file 12954_2026_1456_MOESM1_ESM.docx]

## Supplemental Tables

**Supplemental Table 1: The average grams of dried flower consumed per day, on days used (reported as either joints or g/oz), among people who consume dried flower in the past 12 months in the UK in 2023.**

|  | **People who consumed dried flower in the past 12 months** | **People who consumed dried flower daily in the past 12 months** | **People who consumed dried flower non-daily (weekly, monthly or less than monthly) in the past 12 months** |
| --- | --- | --- | --- |
| Mean | **1.65g**  95% CI: 1.53g – 1.78g  SE=0.065 | **2.69g**  95% CI: 2.42g – 2.96g  SE=0.136 | **1.24g**  95% CI: 1.11g – 1.36g  SE=0.064 |
| 10^th^ quantile | **0.13g**  95% CI: 0.13g – 0.20g  SE=0.019 | **0.30g**  95% CI: 0.20g – 0.50g  SE=0.076 | **0.13g**  95% CI: 0.13g – 0.20g  SE=0.019 |
| 25^th^ quantile | **0.40g**  95% CI: 0.40g – 0.50g  SE=0.025 | **1.00g**  95% CI: 1.00g – 1.80g  SE=0.203 | **0.25g**  95% CI: 0.25g – 0.40g  SE=0.038 |
| 50^th^ quantile (median) | **1.00g**  95% CI: 1.00g – 1.20g SE=0.051 | **2.40g**  95% CI: 2.00g – 3.00g  SE=0.255 | **0.80g**  95% CI: 0.80g – 1.00g  SE=0.051 |
| 75^th^ quantile | **2.40g**  95% CI: 2.00g – 3.00g  SE=0.255 | **3.60g**  95% CI: 3.50g – 4.00g  SE=0.127 | **1.60g**  95% CI: 1.60g – 2.00g  SE=0.102 |
| 90^th^ quantile | **4.00g**  95% CI: 4.00g – 5.00g  SE=0.255 | **6.00g**  95% CI: 5.00g – 7.10g  SE=0.533 | **3.00g**  95% CI: 3.00g – 3.60g  SE=0.153 |
| Minimum | 0.06g | 0.06g | 0.06g |
| Maximum | 7.10g | 7.10g | 7.10g |
| N | 527 | 141 | 386 |

**Supplemental Table 2: Binary logistic regression models of the associations between risk perceptions of different routes of cannabis administration and frequency of cannabis use among people who consume cannabis.**

|  | **Smoking cannabis daily**  **Very low/low risk (vs. Else)**  (n=1,521) | | **Vaping cannabis daily**  **Very low/low risk (vs. Else)**  (n=1,521) | | **Consuming cannabis edibles daily**  **Very low/low risk (vs. Else)**  (n=) | |
| --- | --- | --- | --- | --- | --- | --- |
|  | Univariable analysis | Multivariable analysis | Univariable analysis | Multivariable analysis | Univariable analysis | Multivariable analysis |
|  | OR (95% CI) | AOR (95% CI) | OR (95% CI) | AOR (95% CI) | OR (95% CI) | AOR (95% CI) |
| **Cannabis use frequency** *(vs. less than monthly)* |  |  |  |  |  |  |
| Monthly | 1.21 (0.82, 1.78) | 1.33 (0.90, 1.98) | **1.85 (1.26, 2.70)** | **2.22 (1.49, 3.29)** | 1.36 (0.94, 1.95) | **1.67 (1.14, 2.46)** |
| Weekly | 1.43 (0.94, 2.16) | **1.58 (1.03, 2.42)** | **1.64 (1.09, 2.47)** | **1.91 (1.25, 2.91)** | 1.16 (0.78, 1.73) | 1.33 (0.88, 2.00) |
| Daily | **2.34 (1.61, 3.40)** | **2.43 (1.65, 3.58)** | **2.75 (1.91, 3.95)** | **3.26 (2.24, 4.77)** | **2.01 (1.42, 2.84)** | **2.50 (1.74, 3.58)** |
| **Region**  *(vs. England, excl. London)* |  |  |  |  |  |  |
| London |  | 0.85 (0.61, 1.18) |  | **0.62 (0.44, 0.87)** |  | **0.60 (0.43, 0.82)** |
| Wales |  | 0.72 (0.31, 1.65) |  | **0.40 (0.17, 0.97)** |  | **0.20 (0.08, 0.45)** |
| Scotland |  | 1.65 (0.99, 2.75) |  | 1.17 (0.71, 1.91) |  | 1.38 (0.83, 2.28) |
| Northern Ireland |  | 0.50 (0.21, 1.20) |  | 0.49 (0.20, 1.19) |  | 1.07 (0.44, 2.58) |
| **Sex-at-birth**  *(vs. Female)* |  |  |  |  |  |  |
| Male |  | 0.87 (0.66, 1.16) |  | 0.96 (0.73, 1.27) |  | 0.93 (0.71, 1.22) |
| **Age**  *(vs. 56-65)* |  |  |  |  |  |  |
| 16-25 |  | 1.39 (0.85, 2.27) |  | 1.08 (0.67, 1.74) |  | 0.76 (0.49, 1.17) |
| 26-35 |  | **1.97 (1.17, 3.34)** |  | 1.15 (0.68, 1.92) |  | 0.66 (0.41, 1.07) |
| 36-45 |  | **1.83 (1.13, 2.97)** |  | 1.05 (0.65, 1.69) |  | 0.74 (0.48, 1.15) |
| 46-55 |  | 1.59 (0.92, 2.76) |  | 0.85 (0.49, 1.46) |  | 0.84 (0.51, 1.38) |
| **Ethnicity/race**  *(vs. White)* |  |  |  |  |  |  |
| Asian |  | **0.49 (0.24, 0.98)** |  | 0.60 (0.30, 1.21) |  | 0.60 (0.30, 1.17) |
| Black |  | 0.79 (0.40, 1.56) |  | **0.43 (0.20, 0.96)** |  | 0.55 (0.25, 1.21) |
| Mixed |  | 1.55 (0.77, 3.15) |  | 0.93 (0.50, 1.72) |  | 0.95 (0.52, 1.75) |
| **Highest level of education**  *(vs. Less than high school)* |  |  |  |  |  |  |
| Completed high school |  | 1.64 (0.98, 2.73) |  | 1.41 (0.86, 2.31) |  | 1.12 (0.69, 1.83) |
| Some college or technical vocation |  | 1.03 (0.65, 1.64) |  | 1.02 (0.65, 1.58) |  | 0.81 (0.53, 1.26) |
| Bachelor’s degrees or higher |  | 0.76 (0.47, 1.23) |  | 0.82 (0.52, 1.30) |  | 0.68 (0.43, 1.08) |
| **Income adequacy**  **(vs. very difficult/difficult)** |  |  |  |  |  |  |
| Neither easy nor difficult |  | 1.37 (0.94, 1.99) |  | 1.19 (0.83, 1.73) |  | 1.05 (0.74, 1.50) |
| Very easy/Easy |  | **1.63 (1.12, 2.37)** |  | 1.39 (0.96, 2.03) |  | 1.21 (0.85, 1.73) |
| Unstated |  | 1.71 (0.55, 5.31) |  | 1.20 (0.45, 3.22) |  | 0.99 (0.39, 2.58) |
